# Alpha-bisabolol protects against neonatal asthma by suppressing airway inflammatory signaling

**DOI:** 10.7150/ijms.96371

**Published:** 2025-07-24

**Authors:** Rekha Thiruvengadam, Mydhili Govindarasu, Jamal Mohammed Ali Khaled, Seungho Lee, Jin Hee Kim

**Affiliations:** 1 Centre for Global Health Research-Helix Research Lab, Department of Neonatology, Saveetha Medical College and Hospital, Saveetha Institute of Medical and Technical Sciences (SIMATS), Saveetha University, Chennai, 600077, India.; 2 Department of Integrative Bioscience & Biotechnology, Institute of Bioscience, Sejong University, Seoul 05006, Republic of Korea.; 3 Institute of Forest Science, Kangwon National University, Chuncheon 24341, Republic of Korea.; 4 Department of Botany and Microbiology, College of Science, King Saud University, P. O. Box 2455, Riyadh 11451, Saudi Arabia.; 5 Department of Preventive Medicine, College of Medicine, Dong-A University, Busan 49201, Republic of Korea.; 6 Environmental Health Center, Dong-A University, Busan 49201, Republic of Korea.

**Keywords:** Alpha-bisabolol, Neonatal asthma, Inflammation

## Abstract

**Objective:** This study aimed to evaluate the anti-inflammatory effects of alpha-bisabolol (AB) in allergic airway inflammation-induced rat pups.

**Methods:** We evaluated the anti-adverse effects of AB against allergic airway inflammation-induced male Wistar rat pups, with four categorized groups including vehicle-controls (group 1), controls treated with 25 mg/kg of AB (group 2), allergic airway inflammation-induced cases (group 3), and cases treated with 25 mg/kg of AB before allergic airway inflammation induction (group 4). Lung histopathology, bronchoalveolar lavage fluid eosinophils, and several inflammatory markers were also examined in each group.

**Results:** AB significantly decreased mucous gland hypertrophy, eosinophil infiltration, and oxidative stress marker levels in the allergic airway inflammation-induced AB-pretreated rats. Moreover, AB pretreatment significantly reduced the levels of proinflammatory cytokines, such as interleukin (IL)-1β, IL-6, IL-8, IL-17, monocyte chemoattractant protein-1, C-X-C chemokine receptor type 4 (CXCR4), and thymic stromal lymphopoietin, which were increased in allergic airway inflammation-induced cases. Furthermore, transcription of *cyclooxygenase-2*, *tumor necrosis factor-α*, *CXCR4*, *toll-like receptor 4*, *Eotaxin-1*, and *regulated upon activation normal T cell expressed and secreted* were significantly suppressed in allergic airway inflammation-induced AB-pretreated rats.

**Conclusions:** These results indicate that AB can protect against neonatal asthma by inhibiting acute or chronic inflammation induced during disease onset.

## Introduction

Asthma, a chronic respiratory disease, poses a major health problem in industrialized societies 1,2. Individuals with asthma experience mild to severe breathlessness, chest tightness, and prominent wheezing, which are common symptoms of asthma due to mucus accumulation in the airway passage 3. This causes airway hyperresponsiveness, which narrows the airway passage and smooth muscle cell contraction 4. Inflammation, a major pathological event in asthma, causes airway wall thickening 4. Further, airway smooth muscle cell hypertrophy, increased subepithelial fibrosis, epithelial goblet cells, mucous gland hyperplasia, extracellular matrix degradation, and inflammatory cell infiltration narrow the airways in asthma cases 5-8.

Various studies have experimentally revealed lung inflammation in animal models and developed airway remodeling in the lungs, similar to the effects of asthma in humans 9. The detailed mechanism of airway remodeling through airway inflammation remains unclear, but T-cell and eosinophil recruitment in the lungs 10,11 and lung parenchyma 12 has been accepted as the determining event in the development of asthma.

The importance of T-helper type 2 (Th2) cytokines, such as interleukin (IL)-4, IL-5, IL-9, and IL-13, and chemokines in the pathogenesis of asthma is well known 13. Corticosteroid administration in the form of inhalers has been regularly used by patients with asthma to relieve lung or chest discomfort, release accumulated mucus, and partially decrease airway wall thickness 14. Continuous corticosteroid administration causes side effects 15; hence, herbal medicine-based therapy is advocated 16.

Alpha-bisabolol (AB) is a natural monocyclic sesquiterpene alcohol found in many aromatic plants including black pepper (*Piper nigrum*) and ylang ylang (*Cananga odorata*) with well known anti-inflammatory, antibiotic, analgesic, and anticancer properties 17,18. AB is considered safe because of its low toxicity, as recommended by the Food and Drug Administration, and thus has been used in various commercial products, including cosmetic formulations, as a skin conditioning agent 17. Therefore, the current study evaluated the anti-adverse effects of AB in allergic airway inflammation-induced rat pups developed via allergen exposure, such as ovalbumin (OVA), which mimics the process of allergen exposure observed in human patients with asthma.

## Materials and Methods

### Allergic airway inflammation induction and AB treatment

Twenty-four male Wistar strain rat pups (10 days-old) were used in animal experiments for allergic airway inflammation. All pups were maintained at 20-25 °C with 50-70% relative humidity, fed commercial rat chow, and had free access to clean tap water. Wistar rat pups were categorized into four groups (n=6 for each group): vehicle controls administered normal saline (0.9% NaCl) (group 1), controls treated with 25 mg/kg of AB (group 2), allergic airway inflammation-induced cases (group 3), and cases treated with 25 mg/kg of AB before allergic airway inflammation induction (group 4). Sensitization and OVA treatment were conducted according to the previous procedures with slight modifications 19,20. Briefly, rat pups were sensitized by 0.75% (w/w) OVA inhalation using an ultrasonic nebulizer for 10 min daily for 45 days. Group 4 rats with allergic airway inflammation were orally co-administered AB (25 mg/kg) daily for same 45 days. Group 4 rats with allergic airway inflammation were orally co-administered AB (25 mg/kg). Following all treatments, all rat pups were euthanized via cervical vertebral decapitation. Lung tissue and blood samples were collected for histological and biochemical analyses.

### Histological analyses

Lung tissues were isolated from each rat and fixed in 10% formalin solution for 24 h. Paraffin wax-embedded tissues were cut into 5-µm slices using a microtome and stained with Hematoxylin and Eosin Staining Kit (Abcam Inc., Boston, MA, USA).

### Assessment of eosinophil infiltration and serum Immunoglobulin E levels

Lung fluid was collected to estimate eosinophil infiltration in the bronchoalveolar lavage fluid (BALF), and bronchoalveolar lavage cells were counted using a hemocytometer after staining. IgE levels in the serum were determined using a commercial rat IgE enzyme-linked immunosorbent assay (ELISA) kit (Elabscience, Texas, USA) following the manufacturer's instructions.

### Estimation of oxidative stress markers

Rat lung weights were measured, and the lungs were homogenized in a glass homogenizer using 10 mL/g ice-cold phosphate-buffered saline (PBS, pH 7.5). After centrifuging all samples for 10 min at 4 °C and 12,000 rpm, the supernatants were stored at -80 °C for consecutive assays. Colorimetric assay kits (Nitric oxide assay kit, Protein carbonyl content assay kit, Lipid peroxidation (MDA) assay kit, and Xanthin oxidase activity assay kit (Abcam Inc, Boston, USA)) were used to estimate oxidative stress markers, such as nitric oxide (NO), protein carbonyl content (PCO), malondialdehyde (MDA), and xanthine oxidase (XO) in serum of lung tissue following manufacturers' recommendation 21.

### Detection of inflammation-related signaling molecules

Inflammation-related lipids, such as cysteinyl leukotriene, prostaglandin E2, thromboxane B2, and inflammation-related proteins, including proteoglycan 4 (PRG4) and glycosaminoglycan, were elucidated using commercial lipid assay kits, including Cysteinyl leukotriene ELISA kit (Biomol, Hamburg, Germany), Prostaglandin E2 ELISA kit (Abcam Inc., Boston, MA, USA), Thromboxane B2 ELISA kit (Abcam Inc., Boston, MA, USA), ELISA kit for Proteoglycan 4 (Biozol Diagnostica, Eching, Germany), and total glycosaminoglycan assay kit (Abcam Inc., Boston, MA, USA).

### Serum cytokine level estimation

Proinflammatory cytokines, such as IL-1β, IL-6, IL-8, IL-17, monocyte chemoattractant protein-1 (MCP-1), C-motif chemokine receptor-4 (CCR4), and thymic stromal lymphopoietin (TSLP), and anti-inflammatory cytokines, such as IL-2, IL-9, and IL-13, in serum samples were evaluated using ELISA commercial kits (rat interleukin 8 receptor beta ELISA kit (Biomatik, Delaware, USA), CCR4 ELISA kit (Aviva Systems Biology Corp., California, USA), Rat IL-9 ELISA kit (Abcam Inc., Boston, USA), Rat IL-1β ELISA kit, Rat IL-6 ELISA kit, Rat IL-17 ELISA kit, Rat MCP-1 ELISA kit, mouse thymic stromal lymphopoietin ELISA kit, Rat IL-2 ELISA kit, and Rat IL-13 ELISA kit (Elabscience, Texas, USA)) according to the manufacturer's instructions.

### Reverse transcription-polymerase chain reaction (RT-PCR)

mRNA was extracted from neonatal lung tissues using RNeasy Pure mRNA Bead kit (Qiagen, Hilden, Germany) to elucidate inflammatory signaling activated in allergic airway. A high-capacity cDNA Reverse Transcription kit (Bio-Rad, Hercules, CA, USA) was used to convert 20 µL of mRNA into cDNA. Real-time RT-PCR for specific genes was performed with reverse transcribed cDNA using the SYBR® Green PCR kit (Bio-Rad, Hercules, CA, USA). The Ct values obtained were compared with the control value, and the comparative Ct method (ΔΔCT) was used to determine the fold transcription. *GAPDH*, a housekeeping gene, was used as the internal gene expression control. Primer sequences used are listed in Table 1.

### Statistical evaluation

All data obtained from the experiments were expressed as mean ± standard error of the mean. Comparisons among groups were conducted using analysis of variance (ANOVA) with GraphPad Prism software (Dotmatics, Boston, USA). Significant differences among the groups were determined based on a *p*-value of < 0.05.

## Results

### Histological assessment

**Figure 1:**
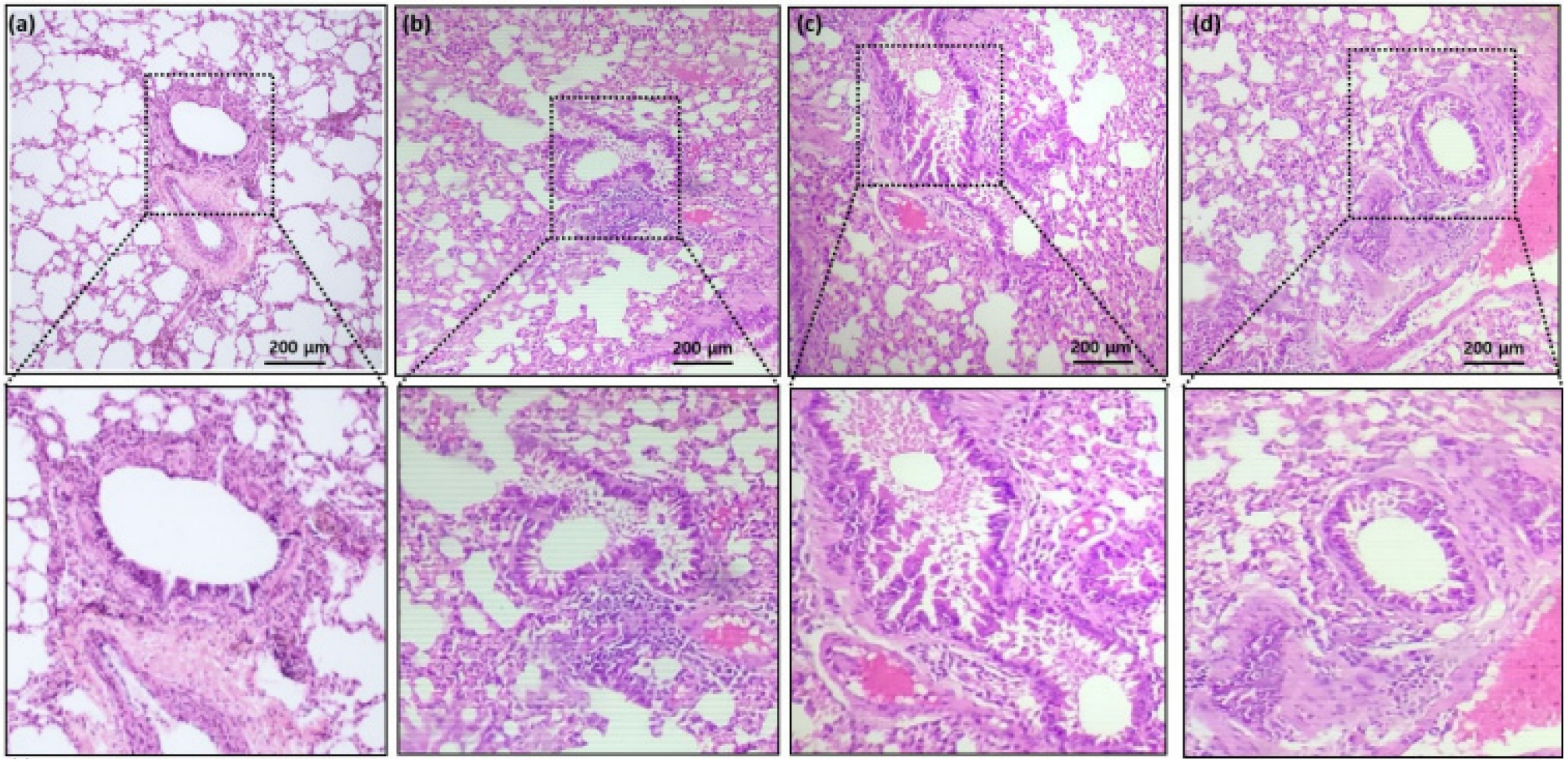
Hematoxylin and eosin (H&E stain) histopathology of rat lung tissue. (a) Group 1 (Control) shows the normal architecture of lung with moderate size of inflammatory cell infiltrates. (b) Group 2 (control treated with AB) shows reduced size of inflammatory cell infiltrates compared with group 1. (c) Group 3 (allergic airway inflammation-induced cases) shows a largest size of inflammatory cell infiltrates. (d) Group 4 (allergic airway inflammation-induced, but treated with AB) shows reduced size of inflammatory cell infiltrates compared with group 3. Black scale bar represents 200 µm.

Compared to the controls (Fig. 1a and 1b), histological assessment of the lung tissues revealed significantly increased vascular congestion, mucous gland hypertrophy, and smooth muscle mass (Fig. 1c). Rats coadministered with AB (Fig. 1d) demonstrated significantly reduced soft muscle mass and hypertrophy development compared to allergic airway inflammation-induced rats (Fig. 1c). OVA sensitization and challenge increased airway reactivity, whereas AB treatment group displayed reduced airway inflammation compared with OVA-sensitized and challenged group or control group.

### Eosinophil infiltration and serum Immunoglobulin E levels

**Figure 2:**
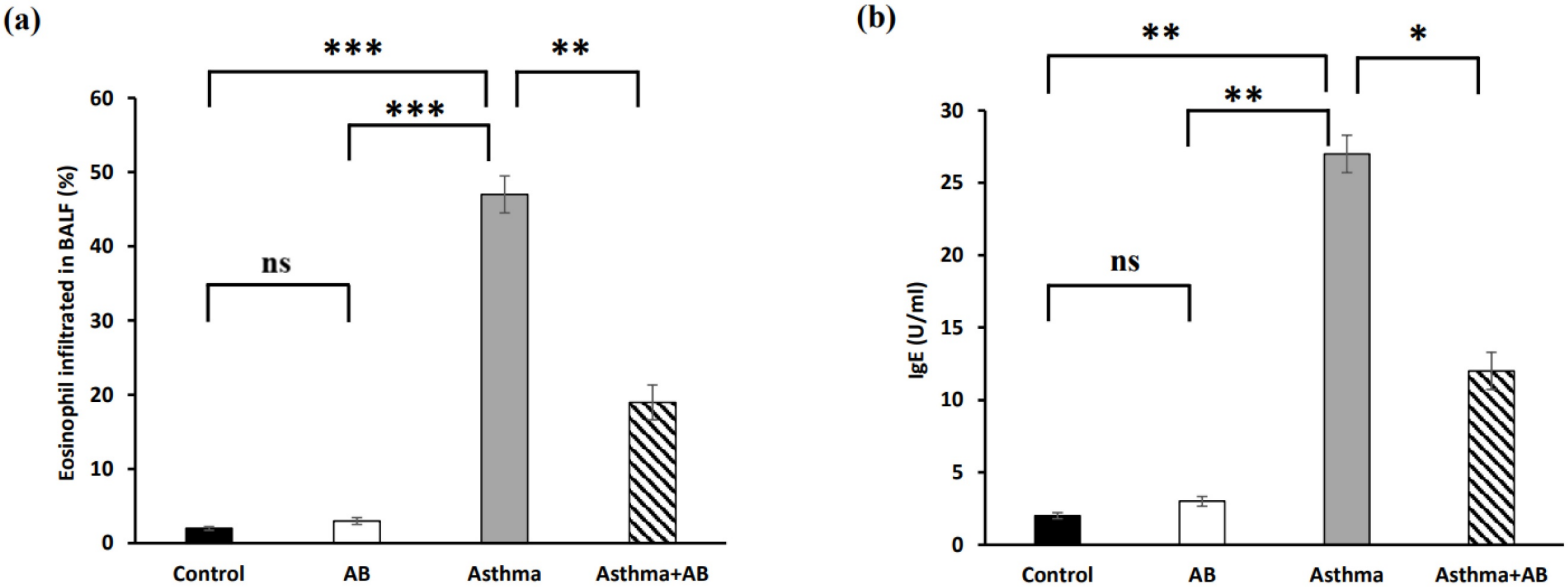
The percent of eosinophils infiltrated in rat BALF and serum IgE level. (a) Eosinophil infiltration in BALF (n = 6 rats/group) and (b) serum IgE (n = 6 rats/group) were calculated. Values were expressed as mean ± SD. Statistical significance was expressed as *p < 0.05, **p < 0.01 and ***p < 0.001. ns, non-significance.

Eosinophil infiltration in BALF significantly increased in allergic airway inflammation-induced rat pups compared to controls, with eosinophils constituting 1.96% of the total leukocytes in normal rat pups and 47% in allergic airway inflammation-induced rat pups (Fig. 2a). This trend was also observed for IgE, with the lowest IgE level (2 U/ml) in normal rat pups and the highest IgE level (27 U/ml) in allergic airway inflammation-induced rat pups (Fig. 2b). Conversely, AB treatment substantially decreased both eosinophil infiltration (19%) and IgE levels (12 U/ml) in allergic airway inflammation-induced rat pups, indicating a protective effect of AB against allergic airway inflammation (Fig. 2).

### Oxidative stress markers

**Figure 3:**
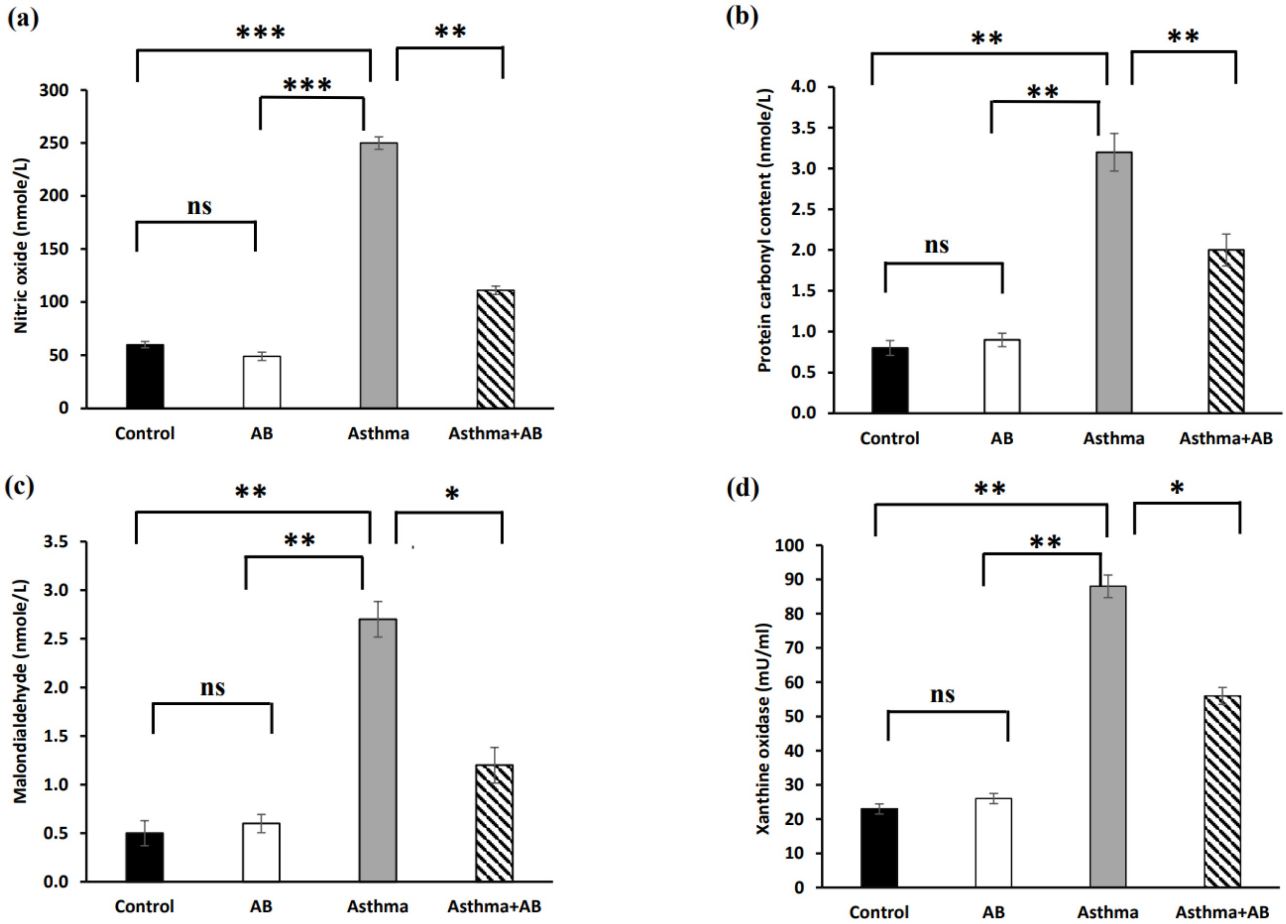
Oxidative stress marker levels in serums of rat lung tissues. (a) Nitric oxide levels, (b) protein carbonyl content, (c) malondialdehyde levels, and (d) xanthine oxidase levels were measured. Values were expressed as mean ± SD. Statistical significance was expressed as *p < 0.05, **p < 0.01 and ***p < 0.001. ns, non-significance.

Oxidative stress indicators, such as NO, PCO, MDA, and XO, were estimated in serum (Fig. 3), and significantly increased levels of all oxidative stress indicators were exhibited in allergic airway inflammation-induced rat pups (250 nmole/L, 3.2 nmole/L, 2.7 nmole/L, and 88 mU/mL, respectively) compared to the controls (60 nmole/L, 0.8 nmole/L, 0.5 nmole/L, and 23 mU/mL, respectively). However, all of these oxidative stress parameters were significantly attenuated in AB-treated allergic airway inflammation-induced rat pups (111 nmole/L, 2 nmole/L, 1.2 nmole/L, and 56 mU/mL, respectively) compared with allergic airway inflammation-induced rat pups.

### Inflammation-related signaling molecules

**Figure 4:**
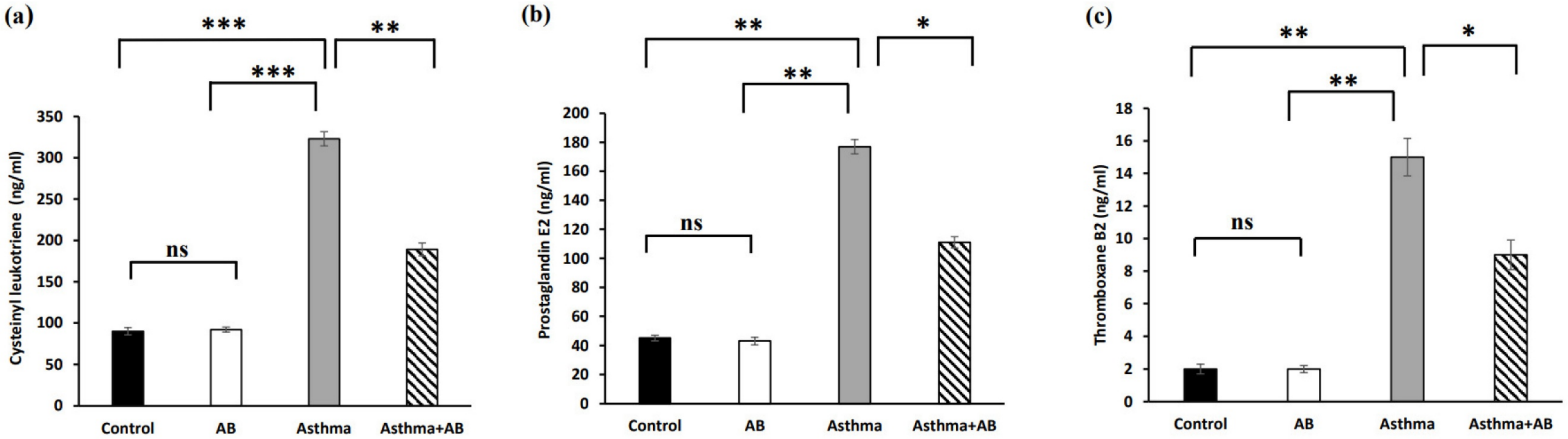
Inflammation-related lipid levels in serums of rat lung tissues. (a) Cysteinyl leukotriene levels, (b) prostaglandin levels, and (c) thromboxane B2 levels were measured. Values were expressed as mean ± SD. Statistical significance was expressed as *p < 0.05, **p < 0.01 and ***p < 0.001. ns, non-significance.

**Figure 5:**
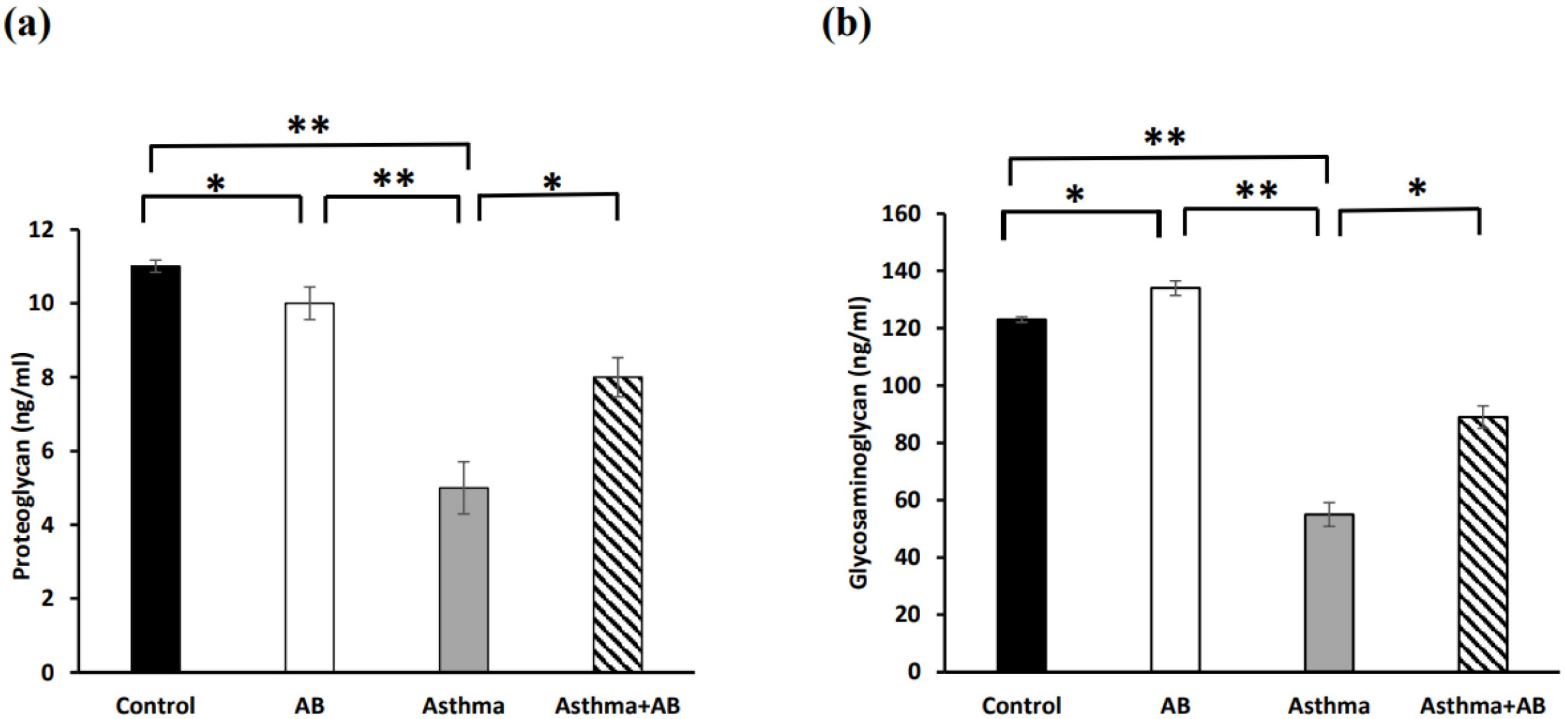
Inflammation-related protein levels in serums of rat lung tissues. (a) Proteoglycan levels and (b) glycosaminoglycan levels were measured. Values were expressed as mean ± SD. Statistical significance was expressed as *p < 0.05, **p < 0.01 and ***p < 0.001. ns, non-significance.

Inflammation-related lipids (cysteinyl leukotriene, prostaglandin E2, and thromboxane B2) and inflammation-related proteins (PRG4 and glycosaminoglycan) were assessed to evaluate the allergic airway inflammation-induced conditions. The results revealed significant increases in all three inflammation-related lipids in the allergic airway inflammation-induced pups (323, 177, and 15 ng/mL, respectively) compared to the control group (90, 45, and 2 ng/mL, respectively) (Fig. 4). However, AB-treated allergic airway inflammation-induced pups displayed an obvious decrease in inflammation-related lipid levels (189, 111, and 9 ng/mL, respectively) compared to allergic airway inflammation-induced rat pups not treated with AB (Fig. 4), indicating the significant protective effects of AB. Furthermore, both inflammation-related protein levels (PRG4 and glycosaminoglycan) were complemented in AB-treated allergic airway inflammation-induced pups (8 and 89 ng/mL, respectively) compared to those in allergic airway inflammation-induced pups (5 and 55 ng/mL, respectively) (Fig. 5). AB supplementation decreased inflammation-related protein levels in allergic airway inflammation-induced pups.

### Estimation of cytokine levels

**Figure 6:**
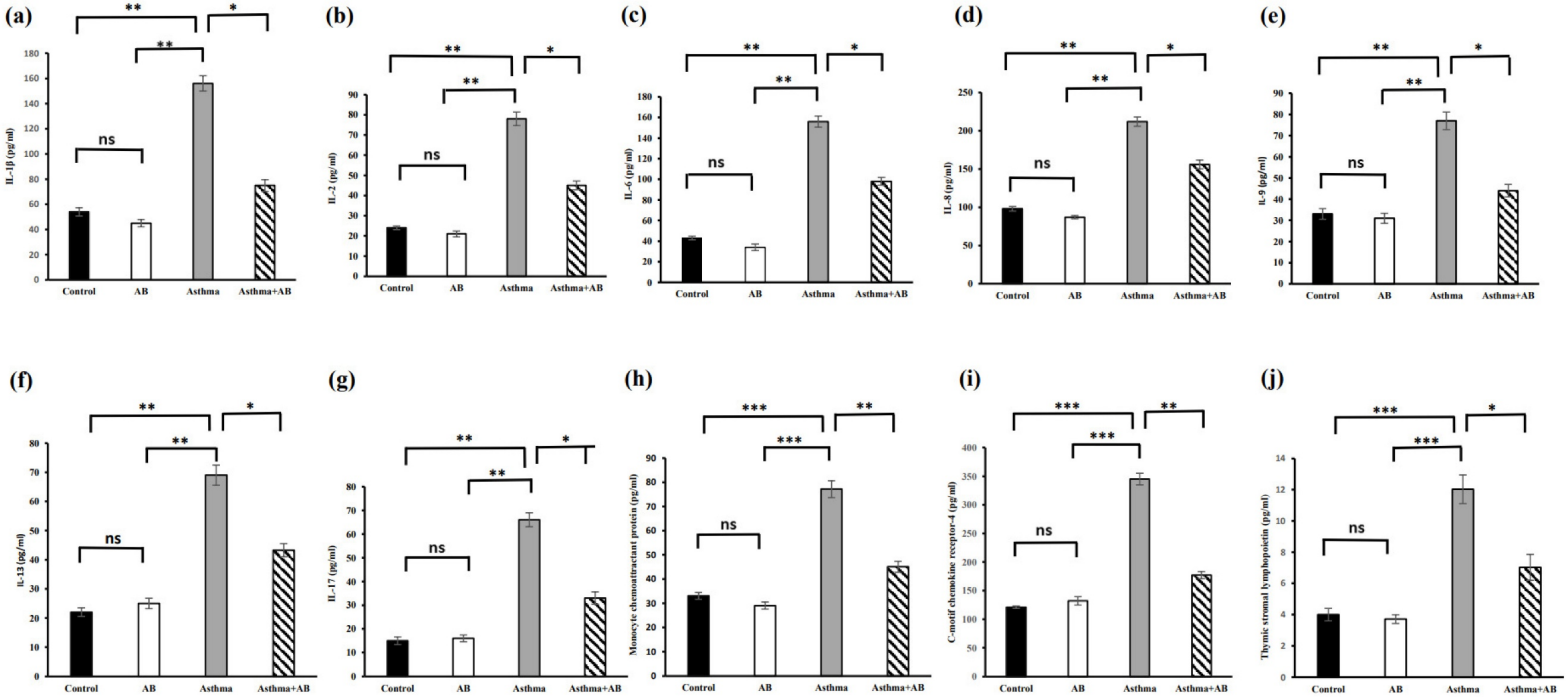
Cytokine levels in serums of rat lung tissues. (a) IL-1β level, (b) IL-2 level, (c) IL-6 levels, (d) IL-8 levels, (e) IL-9 levels, (f) IL-13 levels, (g) IL-17 levels, (h) monocyte chemoattractant protein levels, (i) C Motif Chemokine Receptor 2 levels, and (j) thymic stromal lymphopoietin levels were measured. Values were expressed as mean ± SD. Statistical significance was expressed as *p < 0.05, **p < 0.01 and ***p < 0.001. ns, non-significance.

The serum cytokine levels were also assessed (Fig. 6). Allergic airway inflammation-induced pups showed substantial increases in all proinflammatory cytokines (IL-1β, IL-6, IL-8, IL-17, MCP-1, CCR4, and TSLP at 156, 156, 212, 66, 77.2, 345, and 12 pg/mL, respectively) and anti-inflammatory cytokine levels (IL-2, IL-9, and IL-13 at 78, 77, and 69 pg/mL, respectively) compared to the control (54, 43, 98, 15, 33.1, 121, and 4 pg/mL; and 24, 33, and 22 pg/mL, respectively). However, these cytokine levels were reduced in AB-treated allergic airway inflammation-induced pups (75, 98, 156, 33, 45.1, 177, and 7 pg/mL; and 45, 44, and 43.3 pg/mL, respectively) (Fig. 6), indicating that AB treatment actively suppressed asthma progression, probably by inhibiting the action of inflammatory molecules.

### Fold changes in mRNA levels of inflammation-related genes

**Figure 7:**
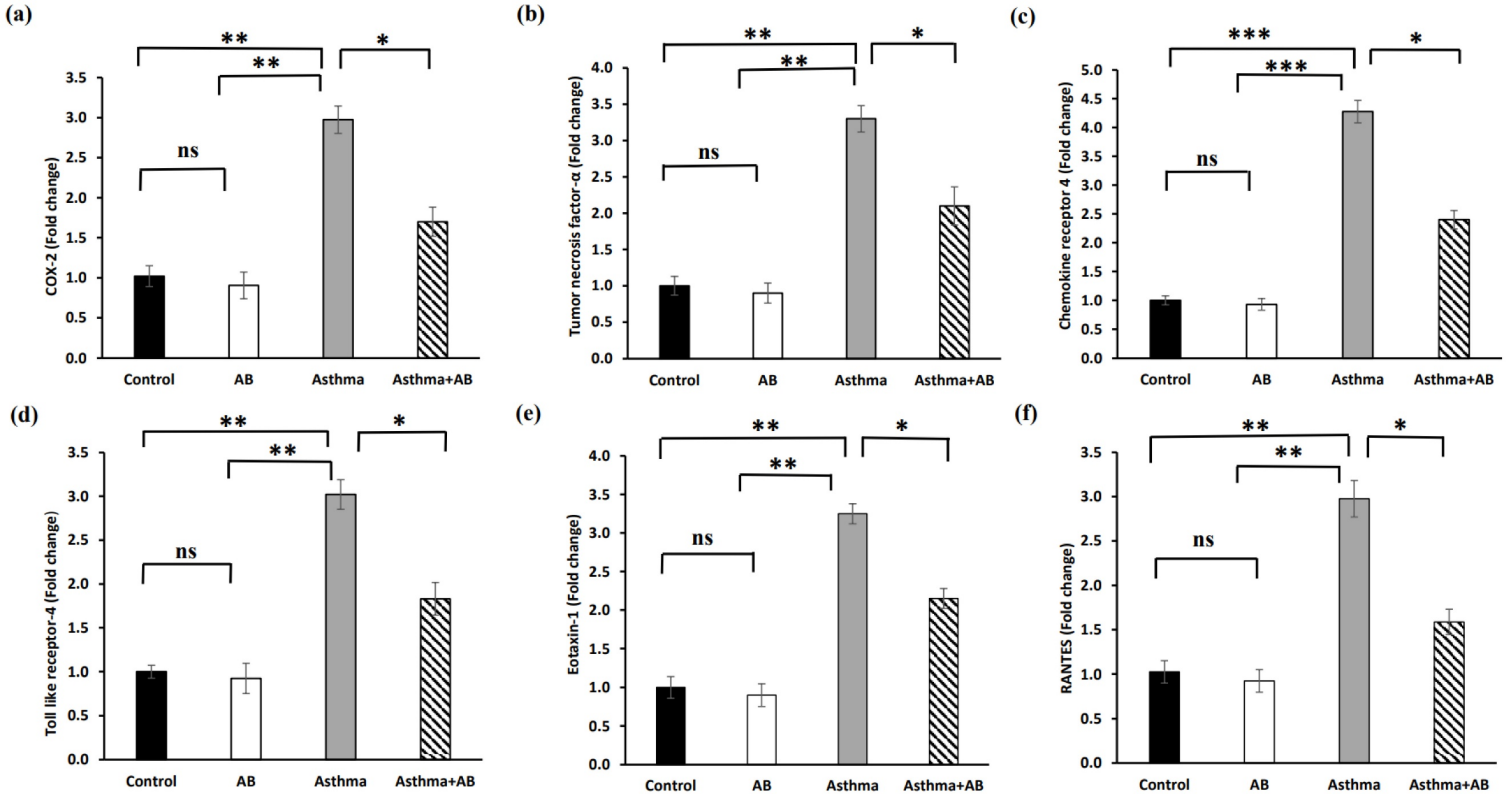
Fold changes in mRNA levels of inflammation-related genes. (a) COX-2 (b) tumor necrosis factor-α, (c) chemokine receptor 4, (d) toll like receptor-4, (e) eotaxin-1, and (f) RANTES were calculated. Values were expressed as mean ± SD. Statistical significance was expressed as *p < 0.05, **p < 0.01 and ***p < 0.001. ns, non-significance.

The transcription levels of inflammation-related genes were determined to substantiate the role of AB in inflammatory signal interruption (Fig. 7). The mRNA expression of *cyclooxygenase-2* (*COX-2*), *tumor necrosis factor-α* (*TNF-α*), *C-X-C chemokine receptor type 4* (*CXCR4*), *toll-like receptor 4* (*TLR4*), *Eotaxin-1*, and *regulated upon activation of normal T-cell expressed and secreted* (*RANTES*) were significantly increased in allergic airway inflammation-induced pups by 2.9-fold, 3.0-fold, 4.2-fold, 3.0-fold, 3.2-fold, and 2.9-fold, respectively, compared to the controls (Fig. 7). However, AB treatment suppressed these transcriptional inflammatory signalings, indicating that AB exerts anti-inflammatory effects against allergic airway inflammation-induced conditions.

## Discussion

Asthma is a chronic inflammatory disease related with airway hyperresponsiveness to various irritants like smoke, dust, pollen, or other allergens 3. This leads to wheezing, due to mucus accumulation in the airway passage, resulting in breathing discomfort 22. The infiltration of immune cells like eosinophils, mast cells, and other leukocytes has been associated with disease pathophysiology, causing inflammation owing to their activation by allergens 22. In our study, OVA sensitization and challenge increased alveolar reactivity in histological assessment and AB reversed it to normal condition. The mucous overproduction, goblet cell hyperplasia, and increased eosinophil infiltration in the peribronchial epithelium were observed in the bronchi, when compared the OVA group to the control group. OVA caused an increase in mucus production, which is driven by the Th2 cytokines IL-4 and IL-13. In particular, IL-13 stimulated mucus-secreting goblet cells in the airway epithelium, while IL-4 promoted mucin gene expression, resulting in mucus hypersecretion 23. The degeneration of alveolar cells, collagen deposition, and goblet cells that secrete mucus around the airway, on the other hand, resulted from the increased inflammation caused by neutrophils, eosinophils, and activated macrophages during the airway inflammation 24. Histological lung sections in our study showed that the AB treatment reduced the amount of mucus in respiratory epithelial cells, goblet cells, and eosinophils, resulting in the degree of inflammation surrounding the bronchus to the levels similar to those in the control group. Previous studies have shown increased infiltration of these cells in the BALF of asthmatic individuals, indicating disease severity that cause subsequent mucus secretion and airway inflammation 25. Significant airway structural remodeling, airway hyperresponsiveness, and airway inflammation can all be exacerbated by early postpartum hyperoxia exposure 25. Airway epithelial barrier integrity can be compromised by reactive oxygen species (ROS), which can also impair cellular function and damage the airway epithelium 26. Ultimately, this can result in an increase in airway smooth muscle, an increase in extracellular interstitial deposition surrounding the airway, and cellular senescence, which can cause airway remodeling 24. Li et al. 27 reported that there was a significant increase in the expression of type 2 cytokines, IL-5 and IL-13, in the BALF of the group treated with O2 and OVA. An increased infiltration of immune cells are also associated with OVA-specific IgE and Th2 cytokine production 28. An increase in airway smooth muscle proliferation has been observed in OVA-exposed animals 29. Treatment with chamomile oil has shown promising effect in reducing the hyperresponsive reaction by decreasing eosinophilic infiltration and IgE levels 30. Our histological analysis indicated that AB treatment could reduce eosinophil infiltration and airway inflammation. Airway inflammation due to immune cell infiltration prompts the production of ROS, leading to oxidative damage to tissues 31. The ROS accumulation induces toxic proteins and lipid peroxidation products 32 and increases endogenous oxidants such as NO, thereby producing NO-derived reactive nitrogen species and XO 33. Superoxide anions produced by eosinophils react with NO to produce reactive nitrogen species, leading to oxidative stress and lung inflammation in OVA-induced asthmatic animals 34,35. Our results revealed significant reductions in stress indicator levels, such as NO, MDA, PCO, and XO, following AB treatment in allergic airway inflammation-induced rats.

Eosinophilia, a characteristic feature of allergic airway inflammation, is associated with increased expression of Th2 cytokines and IgE against OVA 36. Allergens can activate immune cells, such as airway epithelial cells, dendritic cells, alveolar macrophages, smooth muscle, and goblet cells 36. These cells produce the Th2 cytokine IL-13, which is involved in IgE production 37. The current study revealed that AB treatment reduced the inflammatory cells associated with serum IgE expression.

Leukotrienes, produced by activated mast cells and eosinophils 38 are responsible for airway smooth muscle contraction and hyperresponsiveness 39. Cysteinyl leukotrienes, which are potent bronchoconstrictors, are synthesized *de novo* in patients with asthma during an allergic attack, increased in OVA-induced animals, and acted as chemoattractants for eosinophils into the airway mucosa to increase inflammatory process 40. They reduce ciliary motility, hinder mucus clearance, and extrapolate the asthmatic symptoms of wheezing and breathing discomfort by accumulating in the lungs 40. Thromboxane A2, another airway inflammation indicator, contributes to inflammation by thickening and remodeling the airway wall 41. Prostaglandin E2, which is produced by the airway smooth muscles, enhances leukotriene-induced inflammation 42,43, and acts in several ways to increase respiratory inflammation 44. AB treatment reduces eosinophils, effectively reduces leukotriene expression and airway hyperresponsiveness, and inhibits the release of thromboxane B2 and prostaglandin E2 45.

The IgE immune complex (IgE cx) in the acute smooth muscles of asthma triggers an altered airway response, and Th2 cytokines play pivotal roles in asthma pathophysiology 46. IgE immune complexes trigger IL-1β production, a proinflammatory cytokine that mediates airway smooth muscle changes 47. Furthermore, inflammatory cytokines (TNF-α and IL-1β) and Th2 cytokines (IL-5 and IL-13) change the contractile and relaxant responses of airway smooth muscle 48. AB treatment inhibited these responses, decreasing IL-1β expression 49. IL-2 induces calcium release by activating its receptors, resulting in airway smooth muscle contraction 50.

Lin et al. 51 indicated that oxidative stress, various genetic transcriptional regulations, and NF-κB, a proinflammatory transcription factor, induce IL-8, MCP-1, RANTES, eotaxin1, and various proinflammatory cytokines (TNF-α, IL-1β, IL-2, and IL-6) during lung airway inflammation. Another study supported this phenomenon, where the above cytokines were regulated during airway inflammation 52. Our findings showed increased levels of IL-9, IL-13, and IL-17 in allergic airway inflammation-induced rat pups. These cytokines induce Th2 polarization 53. This polarization triggers the degranulation of eosinophils and mast cells, thereby increasing airway hypersensitivity 54. The increased airway hypersensitivity observed in allergic airway inflammation-induced rats was decreased by reducing these inflammatory mediators (IL-9, IL-13, and IL-17) in AB-treated rats, which is associated with the anti-inflammatory effect of AB 55.

Based on our results with previously published knowledge, we summarize that AB exerts its anti-inflammatory effects in neonatal asthma through various mechanisms. (i) AB downregulates the expression and release of proinflammatory cytokines thereby attenuating the inflammatory cascade and reducing airway inflammation 16. (ii) AB inhibits the recruitment and activation of inflammatory cells like eosinophils and T cells, reducing their infiltration into the airways and mitigating airway inflammation and remodeling. (iii) AB interferes with the activity of transcription factors such as NF-κB and AP-1, which control the expression of inflammatory genes, leading to suppression of proinflammatory mediator production and dampening of the inflammatory response 16,17.

## Conclusions

The present study revealed that our animal model was successful in inducing asthmatic symptom, allergic airway inflammation in rats, presenting allergen hyperresponsiveness and airway inflammation in lungs of rat pops. OVA-mediated inflammation in our model was confirmed by increased eosinophil infiltration in the airway smooth muscles. Furthermore, a spike in inflammatory cytokine and chemokine expression activates eosinophils to increase airway hyperresponsiveness in airway inflammation-induced models. Thus, the amelioration of asthma symptoms with reduced airway inflammation is associated with the anti-inflammatory activity of AB. Our results indicate that AB can balance bronchial hematosis. The present *in vivo* study could be a potential approach for using AB as an alternative treatment for asthma. However, further investigation is required to fully understand the pathway and their mechanisms of action. Future research should aim to deepen our understanding of AB's mechanisms of action in neonatal asthma and explore its clinical application as a promising adjunct or alternative treatment option. By advancing our knowledge in this area, we can potentially improve asthma management and enhance the quality of life of pediatric patients with asthma.

## Figures and Tables

**Table 1:** Primers used in polymerase chain reaction.

Gene	Forward primer (5ʹ - 3ʹ)	Reverse primer (5ʹ - 3ʹ)	Annealing temperature (°C)
*COX-2*	CTCAGCCATGCAGCAAATCC	GGGTGGGCTTCAGCAGTAAT	58
*TNF-α*	AAGCTGTCTTCAGGCCAACA	CCCGTAGGGCGATTACAGTC	59
*CXCR4*	GCCATGGCTGACTGGTACTT	CACCCACATAGACGGCCTTT	58
*TLR4*	CCTCGAGTGGGAGGACAAT	TGAGGTTAGAAGCCTCGTGC	59
*Eotaxin-1*	TTTCTTGCACCCCAGCTTTG	AAGGTCACGCAGCAAGATGA	59
*RANTES*	TGCCCACGTGAAGGAGTATT	GGAGTAGGGGGTTGCTCAGT	58
*GAPDH*	GAGCGAGATCCCGTCAAG	ATTTCTCGTGGTTCACACCCA	58

## Abbreviations

AB: Alpha-bisabolol
BALF: bronchoalveolar lavage fluid
CCR4: C-motif chemokine receptor-4
COX-2: cyclooxygenase-2
CXCR4: C-X-C chemokine receptor type 4
IL: interleukin
MCP-1: monocyte chemoattractant protein-1
MDA: malondialdehyde
NO: nitric oxide
OVA: ovalbumin
PCO: protein carbonyl content
PRG4: proteoglycan 4
RANTES: regulated upon activation of normal T-cell expressed and secreted
ROS: reactive oxygen species
RT-PCR: Reverse transcription-polymerase chain reaction
Th2: T-helper type 2
TLR4: toll-like receptor 4
TNF-α: tumor necrosis factor-α
TSLP: thymic stromal lymphopoietin
XO: xanthine oxidase
